# Supplementary material for: X-rays-Induced Bystander Effect Consists in the Formation of DNA Breaks in a Calcium-Dependent Manner: Influence of the Experimental Procedure and the Individual Factor
Source: Biomolecules. 2023 Mar 16;13(3):542. doi: 10.3390/biom13030542 (PMC10046354; doi:10.3390/biom13030542)
Supplement: Supplementary file 1 [file biomolecules-13-00542-s001.zip › biomolecules-2209133-supplementary.pdf]

X-rays-induced bystander effect consists in the formation of DNA breaks in a calcium-dependent manner: influence of the experimental procedure and the individual factor

Juliette Restier-Verlet et al. **Supplementary data**

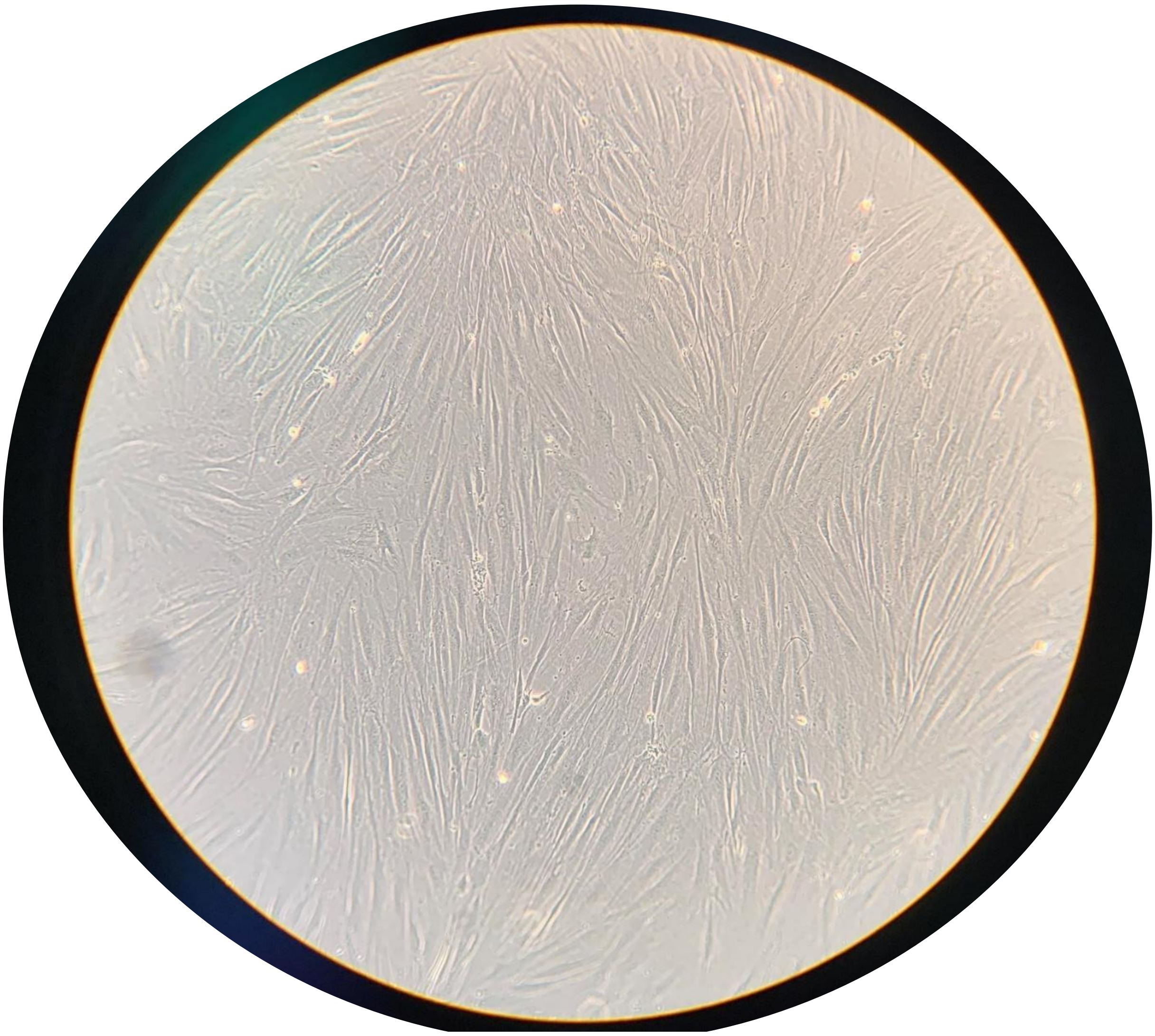

Figure S1: Representative example of an image of fibroblasts monolayer taken from light microscopy (X10).
